# Physical Prehabilitation in Patients who Underwent Major Abdominal Surgery: A Comprehensive Systematic Review and Component Network Meta-Analysis Using GRADE and CINeMA Approach

**DOI:** 10.1245/s10434-023-14632-8

**Published:** 2023-12-01

**Authors:** Claudio Ricci, Laura Alberici, Francesco Serbassi, Paolo Caraceni, Marco Domenicali, Carlo Ingaldi, Davide Giovanni Grego, Carlo Mazzucchelli, Riccardo Casadei

**Affiliations:** 1 Division of Pancreatic Surgery, IRCCS Azienda Ospedaliero-Universitaria Di Bologna, via Albertoni 15, Bologna, Italy; 2 Department of Internal Medicine and Surgery (DIMEC), Alma Mater Studiorum, University of Bologna, Bologna, Italy; 3 Alma Mater Studiorum, Biology of the Health Faculty, University of Bologna, Bologna, Italy; 4 Unit of Semeiotics, Liver and Alcohol-Related Diseases, IRCCS Azienda Ospedaliero-Universitaria of Bologna, Bologna, Italy; 5 Department of Primary Health Care, Internal Medicine Unit Addressed to Frailty and Aging, AUSL Romagna, “S. Maria Delle Croci” Hospital, Ravenna, Italy

**Keywords:** Prehabilitation, Abdominal surgery, Network meta-analysis

## Abstract

**Background:**

Physical prehabilitation is recommended before major abdominal surgery to ameliorate short-term outcomes.

**Methods:**

A frequentist, random-effects network meta-analysis (NMA) was performed to clarify which type of preoperative physical activity among aerobic exercise (AE), inspiratory muscle training (IMT), and resistance training produces benefits in patients who underwent major abdominal surgery. The surface under the P-score, odds ratio (OR), or mean difference (MD) with a 95% confidence interval (CI) were reported. The results were adjusted by using the component network approach. The critical endpoints were overall and major morbidity rate and mortality rate. The important but not critical endpoints were the length of stay (LOS) and pneumonia.

**Results:**

The meta-analysis included 25 studies. The best approaches for overall morbidity rate were AE and AE + IMT (OR = 0.61, p-score = 0.76, and OR = 0.66,* p*-score = 0.68). The best approaches for pneumonia were AE + IMT and AE (OR = 0.21,* p*-score = 0.91, and OR = 0.52,* p*-score = 0.68). The component analysis confirmed that the best incremental OR (0.30; 95% CI 0.12–0.74) could be obtained using AE + IMT. The best approach for LOS was AE alone (MD − 1.63 days; 95% CI − 3.43 to 0.18). The best combination of components was AE + IMT (MD − 1.70; 95% CI − 2.06 to − 1.27).

**Conclusions:**

Physical prehabilitation reduces the overall morbidity rate, pneumonia, and length of stay. The most relevant effect of prehabilitation requires the simultaneous use of AE and IMT.

**Supplementary Information:**

The online version contains supplementary material available at 10.1245/s10434-023-14632-8.

The major surgical procedures produce a significant homeostatic disturbance characterized by catabolism, increased oxygen demand, and inflammatory status.^1^ The stress response is associated with an increased risk of developing postoperative complications.^2^ In recent years, enhanced recovery after surgery (ERAS) has been proposed to reduce the risk of postoperative morbidity. One of the basic concepts of the ERAS approach was that the preoperative period could be used to optimize the overall conditioning of patients.^3^ The preoperative streamlining is called “prehabilitation” and includes correcting modifiable risk factors, such as anemia, malnutrition, smoking, and comorbidities.^4,5^ A not very well-known critical issue of prehabilitation is physical activity. For the patient, facing a major surgery can be compared with “running a marathon.” Thus, similar to a “marathon,” it requires specific and dedicated physical training. Although physical prehabilitation is yet recommended by ERAS guidelines,^4,6,7^ the approach used is not well defined, including a combination of three different techniques: aerobic exercises (AE), such as cycling and walking; resistance training (RT); and inspiratory muscle training (IMT). A recent meta-analysis clearly stated that the best combination for adequate physical prehabilitation is unknown.^8^ Our study was designed to fill this gap by overcoming the multiarm and multicomponent setting problem using a component network meta-analysis (CNMA).^9^ The CINeMA^10^ and GRADE^11^ approaches were used to present the results in an accessible form.

## Material and Methods

The study protocol was preregistered in PROSPERO (CRD42023387987). The manuscript was structured following the Preferred Reporting Items for Systematic Reviews and Meta-Analyses checklist (PRISMA).^12^

### Eligibility Criteria

The eligibility criteria were based on the “Population-Intervention-Control-Outcomes-Studies” (PICOS) approach.^13^ The “Population” was represented by patients who underwent major abdominal surgery, excluding cholecystectomy, abdominal hernia repair, or obesity surgery. The “Intervention” arms considered any physical prehabilitation based on a hospital program. The “Control” group was called nonspecific training (NST) and included any preoperative approach without specific physical prehabilitation. When home activity is recommended without particular exercise, the arm was included the NST group. The “Studies” included only when they reported the morbidity, mortality, or length of stay and only if the design was randomized. CNMA was used to overcome the multiarm problem and isolate the weight of each component.

### Information Source, Search, Study Selection, and Data Collection Process

The research was based on a previous classical meta-analysis,^8^ updating the systematic review. The last search was performed on January 23, 2023. The PubMed/Medline, Embase, and Cochrane databases were used. The search string was managed by using the SR accelerator^14^ and is reported in the Supplementary file.

### Data Items

For each study, we described the first author, year of publication, affiliation, procedures, design (blinded or not), type of disease (malignant or benign), postoperative ERAS management, preoperative nutritional intervention, and compliance rate. The relevance of endpoints was judged by the panel of authors using the GRADE approach (not important, important, critical).^15^ The postoperative morbidity (overall and major) and mortality were considered “critical.” Major morbidity was defined according to the Clavien-Dindo class >2.^16^ The LOS, pneumonia, and postoperative 6-min walking test (6MWT)^17^ were considered “important.” The panel established the minimally important differences as follows^18^: for morbidity (overall and major), mortality, and pneumonia, 10 per 1000 persons more or fewer; for length of stay (LOS), at least ± one day; for 6MWT ± 50 meters. The studies were clustered in different arms based on the AE, RT, and IMT combination.

### Geometries of the Network and Risk of Bias Within the Individual Study

The network geometry was plotted by using nodes and edges. The nodes were the different combinations of interventions, whereas the lines display the observed comparisons: the thickness of the lines is proportional to the number of studies. The frequency component combination was represented by using a dedicated plot.^19^ The risk of bias was based on the revised tool for assessing the risk of bias in randomized trials (Rob2).^20^ The indirectness was considered not negligible when the study population, interventions, and outcomes measurement were not entirely representative of PICOS criteria. Indirectness reduces the transitivity across the common nodes in NMA, returning challenging to obtain credible network estimates. The studies were evaluated as “low-risk,” “some concerns,” or “major concerns.” LA and FS performed Rob2 and indirectness evaluation.

### Summary Measurements and Methods of the Analysis

Frequentist NMA by using random effect was performed. The effect estimates were measured as odds ratios (ORs) or mean differences (MDs), with 95% confidence intervals (CI). The results also were reported as *p*-score, which was the probability, without uncertainty, that combinations would be the best based on the outcome analyzed.^21,22^ The variety of interventions was considered among the best if *p*-score was ≥0.66; when *p*-score was 0.65 to 0.33, the combination was judged inferior to the best/better than the worst; when *p*-score was <0.33, the combination was considered among the worst. The effect of each component intervention also is estimated by CNMA, summing the relative impact of the components comprising this intervention.^9^ The effects of each component were reported as incremental OR (iOR) or MD (iMD) with CI. Results were tabulated according to the GRADE recommendation.^23^ All analysis was made by using the *netmeta and viscomp* package for R version 4.0.1.

### Inconsistency, Risk of Bias Across the Studies, and Meta-Regression Analysis

The global and local incoherence were evaluated.^24^ The local incoherence was related to the unreliability of the networks, and it was described as the ratio of odds ratio (RoR) or difference of MD between direct and indirect estimates. The local incoherence was considered not negligible when the *p*-value was <0.05. The heterogeneity was measured with I^2^.^25^ Publication/reporting bias was investigated using the Begg test.^26^

### Assessment of the Certainty of the Evidence

Based on the GRADE methodology,^27^ four levels of evidence were considered: (1) high quality, which means that the true effect lies close to that of the NMA estimates; (2) moderate quality, which means the actual effect is likely to be close to the NMA estimates, but there is a possibility that it is substantially different; (3) low quality, namely that the true effect may be substantially different from the NMA estimates; (4) shallow quality, which means the true effect is likely to be substantially different from the NMA estimates. The certainty of the evidence was obtained by using online CINeMA software by evaluating the following criteria: within-study bias, reporting bias, indirectness, imprecision, heterogeneity, and incoherence.^28^ If some or major concerns are observed, the certainty is downgraded.

## Results

### Studies Selection, Characteristics, and Risk of Bias Within Studies

**Fig. 1:**
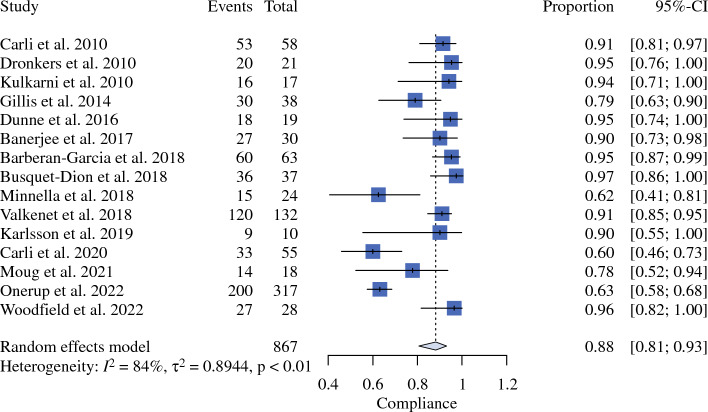
Cumulative proportion of compliance rate

The systematic literature search following the PRISMA statement is reported in Supplementary Fig. 1. In Table 1, the characteristics of the 25 included studies are reported.^29–53^ The details about the prehabilitation programs of included studies are reported in Supplementary Table 1. The patients were clustered into the following intervention arms: Arm NST, including patients preoperative managed without specific training; Arm IMT + AE, including patients who received inspiratory muscle and resistance training; Arm AE + RT, including patients who received aerobic exercise and resistance training; Arm IMT, including patients who received only inspiratory muscle training; Arm AE, including patients who received only aerobic exercise; Arm RT, including patients who received only resistance training; Arm IMT + AE + RT, including patients who received simultaneously aerobic exercise, inspiratory muscle, and resistance training. The meta-analytic compliance results are shown in Fig. 1.

**Table 1:** Characteristics of included studies

First author/year	Components of intervention arm	Affiliation/country	Surgical procedures	Blinded	Malignant/benign disease	Postoperative ERAS approach	Preoperative nutritional intervention	Rob2	Indirectness
Carli et al.^29^	AE + RT	McGill University Health Center, Montreal, Quebec (Canada)	Colorectal surgery	No	Both	Not declared	Not declared	Low risk	Low risk
Dronkers et al.^30^	IMT + AE + RT	Multicentric (Netherlands)	Colorectal surgery	Single	Both	Not declared	Not declared	Some concerns	Low risk
Kulkarni et al.^31^	IMT	Department of Vascular Surgery, Cheltenham General Hospital, Sandford Road, Cheltenham (UK)	Major abdominal surgery	No	Both	Not declared	Not declared	Some concerns	Low risk
Kaibori et al.^32^	AE	Hirakata Hospital, Kansai, Medical University, Osaka (Japan)	Liver resection	No	Malignant	Not declared	Yes	Some concerns	Some concerns
Soares et al.^33^	IMT + AE	Centro de Ciências da Vida, Pontifícia Universidade Católica de Campinas, Campinas, São Paulo (Brazil)	Major abdominal surgery	No	Both	Not declared	Not declared	Low risk	Low risk
Gillis et al.^34^	AE + RT	McGill University, Montreal, Quebec (Canada)	Colorectal surgery	No	Both	Yes	Yes	Low risk	Low risk
Jensen et al.^35^	RT	Institute of Clinical Medicine & Center of Research in Rehabilitation, Aarhus University (Denmark)	Radical cystectomy	Single	Malignant	Yes	Yes	Low risk	Low risk
Dunne et al.^36^	AE	Aintree University Hospital, Longmoor Lane, Liverpool (UK)	Liver resection	Single	Malignant	Not declared	Not declared	Low risk	Low risk
Boden et al.^37^	IMT	Multicentric (Australia and New Zealand)	Major abdominal surgery	Single	Both	Yes	Not declared	Low risk	Low risk
Banerjee et al.^38^	AE + RT	Department of Urology, Norfolk and Norwich University Hospital, Norwich (UK)	Radical cystectomy	Single	Malignant	Yes	Yes	Low risk	Low risk
Barberan-Garcia et al.^39^	AE	Hospital Clinic de Barcelona (Spain)	Major abdominal surgery	Single	Both	Not declared	Yes	Low risk	Some concerns
Busquet-Dion et al.^40^	AE + RT	McGill University Health Center, Montreal (Canada)	Colorectal surgery	Single	Malignant	Yes	Yes	Low risk	Low risk
Minnella et al.^41^	AE + RT	McGill University Health Center, Montreal (Canada)	Esophageal and gastric surgery	Single	Malignant	Yes	Yes	Some concerns	Low risk
Valkenet et al.^42^	IMT	Multicentric (Netherlands)	Esophageal and gastric surgery	Single	Malignant	Yes	Yes	Low risk	Low risk
Ausania et al.^43^	IMT + AE	Complejo Hospitalario Universitario de Vigo (Spain)	Pancreatico duodenectomy	Single	Malignant	Yes	Yes	Some concerns	Low risk
Karlsson et al.^44^	IMT + AE + RT	Stockholm South General Hospital (Sweden)	Colorectal surgery	No	Malignant	Yes	Yes	Some concerns	Some concerns
Carli et al.^45^	AE + RT	McGill University Health Center, Montreal (Canada)	Colorectal surgery	Single	Malignant	Yes	Yes	Low risk	Some concerns
Hernon et al.^46^	AE + RT	Multicentric (UK)	Colorectal surgery	Single	Malignant	Not declared	Not declared	Low risk	Some concerns
Moug et al.^47^	AE	Multicentric (UK)	Colorectal surgery	No	Malignant	Not declared	Not declared	Some concerns	Some concerns
Waller et al.^48^	AE + RT	Colorectal and Peritoneal Oncology Centre, The Christie, Manchester (UK)	Colorectal Surgery	Single	Malignant	Not declared	Yes	Some concerns	Low risk
Allen et al.^49^	AE + RT	Department of General Surgery, The Royal Surrey Hospital, Guildford (UK)	Esophageal and gastric surgery	Single	Malignant	Not declared	Yes	Low risk	Some concerns
Berkel et al.^50^	AE + RT	Multicentric (Netherlands)	Colorectal Surgery	Single	Malignant	Not declared	Yes	Low risk	Some concerns
Gloor et al.^51^	AE + RT	Cantonal Hospital in Winterthur (Switzerland)	Colorectal Surgery	Single	Both	Yes	Not declared	Some concerns	Low risk
Onerup et al.^52^	IMT + AE	Multicentric (Sweden)	Colorectal Surgery	No	Malignant	Yes	Not declared	Low risk	Low risk
Woodfield et al.^53^	AE	Department of Medicine, Dunedin School of Medicine, University of Otago, Dunedin (New Zealand)	Major Abdominal Surgery	Single	Both	Yes	Not declared	Low risk	Low risk

*ERAS* enhanced recovery after surgery; *Rob2* revised tool for assessing the risk of bias in randomized trials; *AE* aerobic exercise; *RT* resistance training; *IMT* inspiratory muscle training

### Network Structures and Geometries and Synthesis of Results

#### Overall Morbidity

**Fig. 2:**
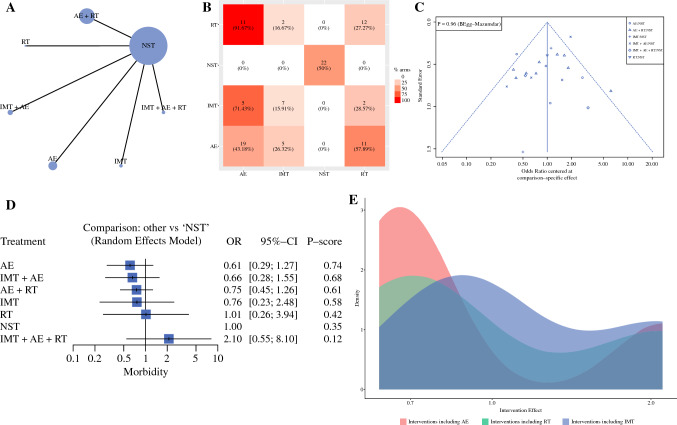
Overall morbidity rate: Network geometry (**A**), Heat plot (**B**), Funnel plot (**C**), Forest plot (**D**), and Density plot (**E**). *AE* aerobic exercise; *RT* resistance training; *IMT* inspiratory muscle training; *NST* no specific training; *OR* odds ratio; *p*-score: the intervention is considered among the best if *p*-score was ≥0.66; when *p*-score was 0.65–0.33, the combination was judged inferior to the best/better than the worst; when *p*-score was <0.33, the combination was considered among the worst

A total of 2404 patients are available for this endpoint. The network geometry is reported in Fig. 2A, whereas the frequency of components is in Fig. 2B. Table 2 shows the results of classical NMA for the morbidity rate. In contrast, Figs. 2C–D show the funnel and forest plots, respectively. Heterogeneity was 50.3%, and global inconsistency was 0.323. Publication bias was absent (Begg test, *p* = 0.960). The assessment of evidence certainty and risk of bias were exhaustively reported in Supplementary Table 2. AE alone (*p*-score = 0.76) and AE + IMT (*p*-score = 0.68) can be considered among the best approaches, reducing the morbidity rate by nearly 1.5 times (OR = 0.61 and OR = 0.66, respectively). The estimated effect was 186 events fewer per 1000 patients treated for AE and 163 events fewer per 1000 patients treated combining AE + IMT. The certainty of the evidence was very low for AE alone for imprecision (95 CI OR include null effect line), significant heterogeneity (*p* = 0.009), and incoherence (conflict results among direct and indirect evidence). The certainty of the evidence was low for AE + IMT due to imprecision (95 CI OR include null effect line) and incoherence (conflict results among direct and indirect evidence), respectively.

**Table 2:** NMA for overall morbidity rate

Total studies: 25 RCT Total Participants: 2404 Inconsistency (τ^2^): 0.323 Heterogeneity (I^2^): 50.3% Test for I^2^ and τ^2^: *p* = 0.009	OR (95% CI)	Anticipated absolute effect^ (95% CrI)			Certainty of the evidence^$^	*p*-score
		With NST*	With intervention	Difference (Minimal important difference = ±10)		
NST	Reference comparator	Reference comparator	Reference comparator	Reference comparator	Reference Comparator	0.35
AE	0.61 (0.29 to 1.27)	478 per 1000	292 per 1000	186 per 1000 fewer (from 340 fewer to 129 more)	⊕◯◯◯, Very Low Indirectness, Imprecision Incoherence	0.76
AE + IMT	0.66 (0.28 to 1.55)	478 per 1000	315 per 1000	163 per 1000 fewer (from 344 fewer to 263 more)	⊕⊕◯◯, Low Imprecision Incoherence	0.68
AE + RT	0.75 (0.45 to 1.26)	478 per 1000	359 per 1000	119 per 1000 fewer (from 263 fewer to 124 more)	⊕⊕◯◯, Low Imprecision Incoherence	0.61
IMT	0.76 (0.23 to 2.48)	478 per 1000	363 per 1000	115 per 1000 fewer (from 368 fewer to 707 more)	⊕⊕◯◯, Low Imprecision Incoherence	0.58
RT	1.01 (0.26 to 3.94)	478 per 1000	483 per 1000	5 per 1000 more (from 354 fewer to 1405 more)	⊕⊕◯◯, Low Imprecision Incoherence	0.42
IMT + AE + RT	2.10 (0.55 to 8.10)	478 per 1000	526 per 1000	48 per 1000 more (from 215 fewer to >9999 more)	⊕◯◯◯, Very Low Indirectness, Imprecision Incoherence	0.12

*OR* NMA estimates are reported as odds ratio; *CI* confidence interval; *CrI* credible interval; *NST* no specific training; *AE* aerobic exercise; *IMT* inspiratory muscle training; *RT* resistance training

^Anticipated absolute effect compares two risks by calculating the difference between the risk of the intervention group with the risk of the control group; the *p*-score represents the probability, without uncertainty, that the approach would be the best

*The baseline morbidity rate was assumed to be those of control group; $ = certainty in evidence according to GRADE working group: (i) High quality—the true effect lies close to that of the estimate of the effect; (ii) Moderate quality—the true effect is likely to be close to the estimate of the effect, but there is a possibility that it is substantially different; (iii) Low quality—the true effect may be substantially different from the estimate of the effect; (iv) Very low quality—the true effect is likely to be substantially different from the estimate of effect; ⊕ Presence of a factor leading to downgrade; ◯ Absence of a factor leading to downgrade

The component analysis (Fig. 2E) showed that none of the components have significant incremental effect: AE, iOR = 0.61 (95% CI 0.35–1.07), *p* = 0.090; RT, iOR = 1.31 (95% CI 0.69–2.47), *p* = 0.404; IMT, iOR = 1.18 (95% CI 0.60–2.30), *p* = 0.902. Moreover, none of the combinations has a statistically significant incremental effect.

### Major Overall Morbidity (CDC > 2)

The morbidity, according to the CDC, is extractable only from 1312 patients. The network geometry is reported in Supplementary Fig. 2A, whereas the frequency of components is in Supplementary Fig. 2B. None of the intervention arms was superior to NST (Supplementary Table 3 and Supplementary Fig. 2C–D). Heterogeneity (0%, *p* = 0.423), global inconsistency (0.001), and publication bias (Begg test, *p* = 0.790) were absent. The detail about the certainty of the evidence is in Supplementary Table 4. The component analysis showed that none of the components or possible combinations has a significant incremental effect. The density plot was not created due to the small number of studies.

### Mortality

A total of 2674 patients are available for this endpoint. The network geometry is reported in Supplementary Fig. 3A, and the frequency of components is in Supplementary Fig. 3B. Supplementary Table 5 shows the results of classical NMA for the morbidity rate. In contrast, Supplementary Figs. 3C and D showed the forest and funnel plots, respectively. Heterogeneity (0%; *p* = 0.986), global inconsistency (tau^2^ = 0), and publication bias (Begg test, *p* = 0.920) were absent. The evidence certainty and risk of bias were exhaustively reported in Supplementary Table 6. None approach significantly reduced the mortality rate. The component analysis showed that none of each component or the possible combinations have a significant incremental effect, as reported in the density plot (Supplementary Fig. 3E).

### Length of Stay

**Fig. 3:**
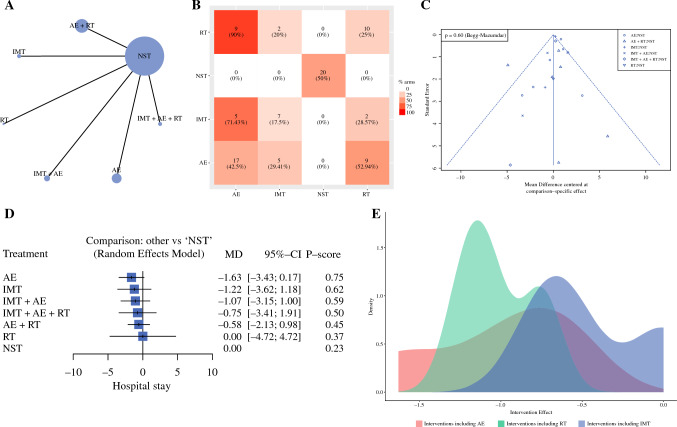
Length of stay: Network geometry (**A**), Heat plot (**B**), Funnel plot (**C**), Forest plot (**D**), and Density plot (**E**). *AE* aerobic exercise; *RT* resistance training; *IMT* inspiratory muscle training; *NST* no specific training; *MD* mean difference; *p*-score: the intervention is considered among the best if *p*-score was ≥0.66; when *p*-score was 0.65–0.33, the combination was judged inferior to the best/better than the worst; when *p*-score was <0.33, the combination was considered among the worst

A total of 2468 patients are available for this endpoint. The network geometry is reported in Fig. 3A, whereas the frequency of components is shown in Fig. 3B. Table 3 shows the results of classical NMA. Figure 3C and D show the funnel and forest plots, respectively. Heterogeneity was 51.8%, and global inconsistency was 1.86. Publication bias was absent (Begg test, *p* = 0.600). The evidence certainty and risk of bias were reported in Supplementary Table 7. The only intervention arm among the best approaches was AE alone (*p*-score = 0.75). Patients treated with AE have a length of stay (LOS) of nearly 2 days inferior to those not treated (MD = − 1.63). The certainty of the evidence was very low for imprecision (95 CI OR include null effect line), significant heterogeneity (*p* = 0.010), and incoherence (conflict results among direct and indirect evidence).

**Table 3:** NMA for the length of stay

Total studies: 20 RCT Total Participants: 2674 Inconsistency (τ^2^): 1.86 Heterogeneity (I^2^): 51.8% Test for I^2^ and τ^2^: *p* = 0.010	MD (95% CI)	Anticipated absolute effect^ (95% CrI)			Certainty of the evidence^$^	*p* score
		With NST*	With intervention	Difference (Minimal important difference = ±1)		
NST	Reference comparator	Reference comparator	Reference comparator	Reference comparator	Reference Comparator	
AE	− 1.63 (− 3.43 to 0.18)	10 days	8 days	2 days fewer (from 3 fewer to 0)	⊕⊕⊕◯, Very low Imprecision, Incoherence, Heterogeneity	0.75
IMT	− 1.22 (− 3.62 to 1.18)	10 days	9 days	1 day fewer (from 4 fewer to 1 more)	⊕⊕◯◯, Low Imprecision, Incoherence	0.62
IMT + AE	− 1.07 (− 3.15 to 1.00)	10 days	9 days	1 day fewer (from 3 fewer to 1 more)	⊕⊕◯◯, Low Imprecision, Incoherence	0.59
IMT + AE + RT	− 0.75 (−3.41 to 1.91)	10 days	9 days	1 day fewer (from 3 fewer to 2 more)	⊕⊕⊕◯, Very low Within-study bias Imprecision, Incoherence, Indirectness	0.50
AE + RT	-0.58 (-2.13 to 0.98)	10 days	19 days	1 days fewer (from 2 fewer to 1 more)	⊕⊕⊕◯, Very low Within-study bias Imprecision, Incoherence, Heterogeneity	0.45
RT	0 (− 4.72 to 4.72)	10 days	10 days	0 (from 5 fewer to 5 more)	⊕⊕◯◯, Low Imprecision, Incoherence	0.37

*MD* NMA estimates are reported as mean difference; *CI* confidence interval; *CrI* credible interval; *NST* no specific training; *AE* aerobic exercise; *IMT* inspiratory muscle training; *RT* resistance training

^Anticipated absolute effect compares two risks by calculating the difference between the risk of the intervention group with the risk of the control group; the p-score represents the probability, without uncertainty, that the approach would be the best

*The baseline morbidity rate was assumed to be those of control group; $ = certainty in evidence according to GRADE working group: (i) High quality—the true effect lies close to that of the estimate of the effect; (ii) Moderate quality—the true effect is likely to be close to the estimate of the effect, but there is a possibility that it is substantially different; (iii) Low quality—the true effect may be substantially different from the estimate of the effect; (iv) Very low quality—the true effect is likely to be substantially different from the estimate of effect. ⊕ Presence of a factor leading to downgrade; ◯ Absence of a factor leading to downgrade

The component analysis showed that no component alone had a significant incremental effect: AE, iMD = − 0.99 (95% CI − 2.08 to 0.09), *p* = 0.075; IMT, iMD = − 0.41 (95% CI − 1.51 to 0.68), *p* = 0.460; RT, iOR = 0.56 (95% CI − 0.79 to 1.93), *p* = 0.415. On the contrary, AE + IMT was the only combination that guaranteed a significant reduction of LOS of nearly 2 days (iMD = − 1.7 days; −2.06 to − 1.27 CI; *p* < 0.001), as shown in Fig. 3E.

### Pneumonia

**Fig. 4:**
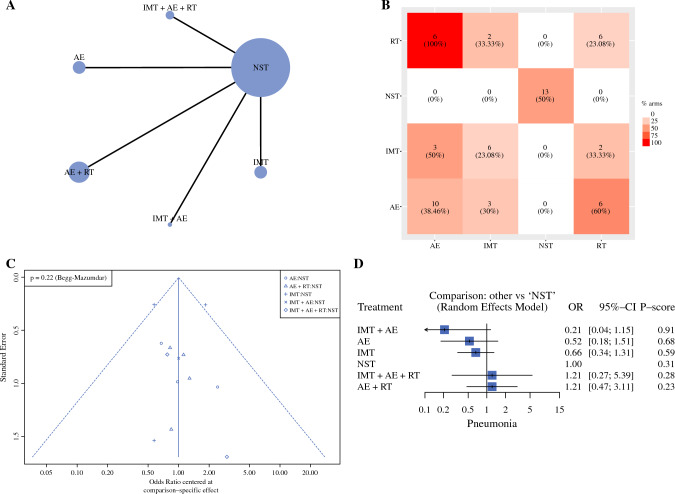
Pneumoniae rate: Network geometry (**A**), Heat plot (**B**), Funnel plot (**C**), and Forest plot (**D**). *AE* aerobic exercise; *RT* resistance training; *IMT* inspiratory muscle training; *NST* no specific training; *OR* odds ratio; *p*-score: the intervention is considered among the best if *p*-score was ≥0.66; when *p*-score was 0.65–0.33, the combination was judged inferior to the best/better than the worst; when *p*-score < 0.33, the combination was considered among the worst

A total of 1452 patients are available for this endpoint. The network geometry is reported in Fig. 4A, whereas the frequency of components is shown in Fig. 4B. Table 4 shows the results of classical NMA for the pneumonia rate. In contrast, Fig. 4C and D show the funnel and forest plots, respectively. Heterogeneity was 34.8% (*p* = 0.139), and global inconsistency was 0.431. Local inconsistency was tested due to the absence of closed loops. Publication bias was absent (Begg test, *p* = 0.220). The assessment of evidence certainty and risk of bias were exhaustively reported in Supplementary Table 8. AE + IMT alone (*p*-score = 0.91) and AE (*p*-score = 0.68) can be considered among the best approaches. AE + IMT reduced the risk of pneumonia (OR = 0.21), whereas AE (OR = 0.52) about half. The expected pneumonia rate was 138 fewer per 1000 patients when AE + IMT was administrated during the preoperative period. The expected pneumonia rate was 84 fewer per 1000 patients when AE was administrated in the preoperative period. The certainty of the evidence was low for AE + IMT due to imprecision (95 CI OR include null effect line) and incoherence (conflict results among direct and indirect evidence). The certainty of the evidence was very low for AE due to imprecision (95 CI OR include null effect line), significant heterogeneity (*p* = 0.010) and incoherence (conflict results among direct and indirect evidence). The component analysis showed that none of the components had a significant incremental effect: AE, iOR = 0.45 (95% CI 0.19–1.06), *p* = 0.066; IMT, iOR = 0.67 (95% CI 0.40–1.11), *p* = 0.119; RT, iOR = 3.02 (95% CI 0.97–9.41), *p* = 0.056.

**Table 4:** NMA for pneumonia rate

Total studies: 14 RCT Total Participants: 1452 Inconsistency (τ^2^): 0.431 Heterogeneity (I^2^): 34.8% Test for I^2^ and τ^2^: *p* = 0.139	OR (95% CrI)	Anticipated absolute effect^ (95% CrI)			Certainty of the evidence^$^	*p*-score
		With NST*	With intervention	Difference (Minimal important difference = ±10)		
NST	Reference comparator	Reference comparator	Reference comparator	Reference comparator	Reference Comparator	0.31
AE + IMT	0.21 (0.04 to 1.15)	175 per 1000	37 per 1000	138 per 1000 fewer (from 168 fewer to 26 more)	⊕⊕◯◯, Low Imprecision Incoherence	0.91
AE	0.52 (0.18 to 1.51)	175 per 1000	91 per 1000	84 per 1000 fewer (from 143 fewer to 89 more)	⊕◯◯◯, Very Low Indirectness, Imprecision Incoherence	0.68
IMT	0.66 (0.34 to 1.31)	175 per 1000	116 per 1000	60 per 1000 fewer (from 116 fewer to 54 more)	⊕⊕◯◯, Low Imprecision Incoherence	0.59
IMT + AE + RT	1.21 (0.27 to 5.39)	175 per 1000	138 per 1000	37 per 1000 more (from 128 fewer to 768 more)	⊕◯◯◯, Very Low Whitin-study bias, Imprecision Incoherence	0.28
AE + RT	1.21 (0.47 to 3.11)	175 per 1000	211 per 1000	37 per 1000 more (from 93 fewer to 369 more)	⊕◯◯◯, Very Low Indirectness, Imprecision Incoherence	0.23

*OR* NMA estimates are reported as odds ratio; *CrI* credible interval; *NST* no specific training; *AE* aerobic exercise; *IMT* Inspiratory muscle training; *RT* resistance training

^Anticipated absolute effect compares two risks by calculating the difference between the risk of the intervention group with the risk of the control group; the p-score represents the probability, without uncertainty, that the approach would be the best. *The baseline morbidity rate was assumed to be those of control group; $ Certainty in evidence according to GRADE working group: (i) High quality—the true effect lies close to that of the estimate of the effect; (ii) Moderate quality—the true effect is likely to be close to the estimate of the effect, but there is a possibility that it is substantially different; (iii) Low quality—the true effect may be substantially different from the estimate of the effect; (iv) Very low quality—the true effect is likely to be substantially different from the estimate of effect. ⊕ Presence of a factor leading to downgrade; ◯ Absence of a factor leading to downgrade

The calculated effect showed that AE + IMT was the only combination with a statistically significant effect. Combining AE + IMT, the pneumonia rate can be significantly reduced of three times: iOR of 0.30 (95% CI 0.12–0.74; *p* = 0.014).

#### 6MWT

CNMA was not performed because, in 21 studies, this datum was not extractable or not reported.

## Discussion

The present study attempts to clarify the role of physical prehabilitation before major abdominal surgery, specifying the weight of each exercise. The study included 25 randomized, controlled trials and 2674 patients, resulting in the largest meta-analysis. The main problem was that the interventional arms were a combination of three different types of supervised physical activity: AE, such as cycling and walking; RT, using elastic bands for different muscles; and IMT device-assisted. This problem was solved by using the NMA approach, while the CNMA methodology was used to weigh each component’s relevance and obtain the plausible best combinations.^9^ Moreover, CINeMA^10,28^ and GRADE^11^ approaches were used to overcome the simple evaluation of statistical significance. The quality of the evidence was evaluated, not only considering the imprecision (namely the statistical significance of effect size) but also testing within-study bias, reporting bias, indirectness, heterogeneity, and incoherence. The first exciting finding derived from the descriptive analysis: the meta-analytic compliance rate was not so low (88%), as suggested by several authors.^41,45,52^ However, the high degree of heterogeneity (I^2^ = 84%) suggested a high variability among the studies depending on the hospital setting, type of surgery, and patients. Concerning morbidity, NMA provided some interesting information. First, AE and AE + IMT arms reduced the overall morbidity rate compared with NST. However, this effect remains uncertain due to imprecision, because the confidence interval crosses the minimal important difference. Moreover, the impact could be highly variable for the AE arm due to indirectness. Indeed, three studies^32,39,47^ enrolled only frailty subjects such as cirrhotic or high-risk patients. The estimated effect suggested a slight advantage for AE alone, even if none of the components or combinations has a statistically significant incremental effect. In other words, the overall complications represent a composite endpoint in which many complications are included, but only a few could be influenced by preoperative exercise. Analysis of major morbidity and mortality confirms this hypothesis. Complications requiring surgical, endoscopic, or radiological reintervention depended on surgical-related factors, such as anastomotic leak, septic events, or hemorrhage. All of these complications cannot be avoided by physical prehabilitation. Physical preoperative exercise could make patients more resistant to the potentially negative consequences of a complicated postoperative course. Still, this effect is challenging to capture by measuring the severity of complications or crude mortality rate. It could be recognized by estimating the failure to rescue rate (FTR), which represents the mortality rate calculated only among patients who experience major complications and depends on several factors, including the preexistent conditions of the patients.^54^ Unfortunately, none of the included studies reported FTR. Considering the pneumonia rate, AE + IMT and AE was the best approach with a clinically relevant effect. However, the imprecision, incoherence not evaluable, and indirectness (only for the AE arm) reduced the certainty of the results. Interestingly, the component analysis confirmed that the best plausible and statistically significant effect could be obtained by combining AE with IMT. These results did not surprise us: it is physiologically reasonable that increasing VO_2_ max and ventilatory capacity could avoid infectious respiratory complications after major abdominal surgery. A recent systematic review indicates that a significant percentage, up to 28%, of major abdominal surgical procedures could be complicated by pneumonia.^55^ The risk increases two or three times when poor lung function is preoperative present. Concerning LOS, the only approach among the best is the AE alone, whereas IMT alone and IMT + AE maintained a marginal effect, resulting in better than worst. These data are uncertain due to a miscellanea of bias, such as imprecision, incoherence, within-study bias, and heterogeneity. The component analysis suggested that the best combination could be AE + IMT with a potentially significant reduction of LOS up to 2 days. These results are credible from a physiopathological point of view, but they should be interpreted with prudence, because the LOS is a weak measure of efficacy. Several confounding factors, such as the age of patients, the type of healthcare system, the type of surgery, and other social factors, easily influence hospitalization. Other objective measures could be used to capture the effect of prehabilitation on the LOS, such as functional recovery, type of discharge (e.g., at home or with the need for further rehabilitation), or quality-of-life questionnaire. Unfortunately, these data are little or nothing available within the included studies.

The present study has some limitations. First, the included studies cover a relatively long period. Another limitation is the lack of a standardized definition of the outcomes not corrigible with rigorous data extraction (within-study bias). These biases can be limited by using all statistical instruments to capture the heterogeneity, inconsistency, and publication bias. The CINeMA approach measured the weight of bias, but they did not erase it. Finally, the local incoherence cannot be evaluated due to the absence of closed loops. Therefore, all studies were considered by default at risk using CINeMA and GRADE methodology.

## Conclusions

The present study suggests that physical prehabilitation could play a role in patients who underwent major abdominal surgery, reducing minor or respiratory complications. This hypothesis seems to be coherent with the observed reduction of LOS, quantifiable in less than 2 days. Even if the ideal approach does not exist, CNMA indicates that the simultaneous use of AE and IMT could be more effective than AE or IMT alone in obtaining clinically relevant results. Further high-quality, randomized studies are needed to validate the routine use of physical prehabilitation. Indeed, physical prehabilitation could reduce the FTR, making the patients more resistant to the negative effects of major complications. Unfortunately, the FTR was never reported, and this remained only a speculative hypothesis. Nonetheless, our results could help design new trials, indicating the simultaneous use of AE + IMT in the intervention arm and the use of new outcomes, such as failure to rescue or functional recovery.

### Supplementary Information

Below is the link to the electronic supplementary material.

Supplementary file1 (DOCX 358 kb)
