# Supplementary material for: Physical Prehabilitation in Patients who Underwent Major Abdominal Surgery: A Comprehensive Systematic Review and Component Network Meta-Analysis Using GRADE and CINeMA Approach
Source: Ann Surg Oncol. 2023 Dec 1;31(3):1725–38. doi: 10.1245/s10434-023-14632-8 (PMC10838229; doi:10.1245/s10434-023-14632-8)
Supplement: Supplementary file 1 — Supplementary file1 (DOCX 358 kb) [file 10434_2023_14632_MOESM1_ESM.docx]

**Methods**

The search string was the following: (("Physical Conditioning, Human"[Mesh] OR "Preoperative Exercise"[Mesh] OR pre-habilitation[tw] OR prehabilitation[tw] OR "pre-operative conditioning"[tw] OR "preoperative conditioning"[tw] OR "physical conditioning"[tw] OR "inspiratory muscle training"[tw] OR "aerobic exercise"[tw] OR "resistance training"[tw]) AND ("General Surgery"[Mesh] OR "Surgical Procedures, Operative"[Mesh] OR "Surgery"[sh] OR "major abdominal surgery"[tw])) AND ("Postoperative Period"[Mesh] OR "Postoperative Complications"[Mesh] OR "Postoperative Care"[Mesh] OR "postoperative outcome*"[tw]). The string was built by combining keywords and MeSH and using the following three concepts: pre-habilitation, major abdominal surgery, and postoperative outcomes.

**Results**

The selection process of the study is plotted in **Supplementary Figure 1.** All studies were conducted in western countries, but only 7 (28%) were multicentric. The study reported several types of abdominal surgical procedures: 12 (48 %) colorectal surgery, 5 (20%) miscellanea di major abdominal procedures, 3 (12%) esophageal or gastric resection, 2 (8%) liver resection, 2 (8%) cystectomies, 1 (4%) pancreatic resection. Seventeen studies (68%) were single-blinded, and the remaining 8 (32%) were open-label. In most studies, only malignant diseases were treated (16, 64%). Four (16%) studies included only frailty patients, 2 (8%) studies only patients treated with neoadjuvant, and 1 (4%) cirrhotic patient. These seven studies were the sources of indirectness. Preoperative nutritional status was evaluated and corrected in 14 (56%) studies, while preoperative physiological counselling was performed in 5 (20%) studies. ERAS approach was declared in 13 (52%) studies. The details of physical activity in the intervention arms were reported in **Supplementary Table1**

**Supplementary Table 1- Details of pre-habilitation programs**

| **Study** | **Intervention Arms** | **Duration, weeks** | **Session number, n/week** | **Session length, min** | **Supervision** | **Optimal Target** |
| --- | --- | --- | --- | --- | --- | --- |
| Carli et al. 2010 ^29^ | AE + RT | 4 | 7 | 45 | Yes | Yes |
| Dronkers et al. 2010 ^30^ | IMT + AE + RT | 2 | 7 | 60 | Yes | Yes |
| Kulkarni et al. 2010 ^31^ | IMT | 2 | 14 | 15 | Yes | Yes |
| Kaibori et al. 2013 ^32^ | AE | 4 | 3 | 60 | Yes | Yes |
| Soares et al. 2013 ^33^ | IMT + AE | 3 | 6 | 50 | Yes | Yes |
| Gillis et al. 2014 ^34^ | AE + RT | 4 | 3 | 50 | Yes | Yes |
| Jensen et al. 2014 ^35^ | RT | 2 | 2 | 15 | Yes | No |
| Dunne et al. 2016 ^36^ | AE | 4 | 3 | 30 | No | Yes |
| Boden et al. 2017 ^37^ | IMT | 6 | 1 | 30 | No | No |
| Banerjee et al. 2017 ^38^ | AE + RT | 4 | 2 | 60 | Yes | Yes |
| Barberan-Garcia et al. 2018 ^39^ | AE | 4 | 3 | 60 | Yes | No |
| Busquet-Dion et al. 2018 ^40^ | AE + RT | 1 | 7 | 60 | Yes | Yes |
| Minnella et al. 2018 ^41^ | AE + RT | 3 | 3 | 60 | Yes | Yes |
| Valkenet et al. 2018 ^42^ | IMT | 2 | 14 | 10 | No | No |
| Ausania et al. 2019 ^43^ | IMT + AE | NR | 7 | 60 | Yes | Yes |
| Karlsson et al. 2019 ^44^ | IMT + AE + RT | 2 | 3 | 60 | Yes | Yes |
| Carli et al. 2020 ^45^ | AE + RT | 4 | 7 | 60 | Yes | No |
| Hernon et al. 2021 ^46^ | AE + RT | 4 | 3 | 45 | Yes | Yes |
| Moug et al. 2021 ^47^ | AE | 13 | 1 | 30 | Yes | Yes |
| Waller et al. 2021 ^48^ | AE + RT | 4 | 3 | 30 | Yes | Yes |
| Allen et al. 2022 ^49^ | AE + RT | 15 | 2 | 60 | Yes | Yes |
| Berkel et al. 2022 ^50^ | AE + RT | 3 | 3 | 60 | Yes | No |
| Gloor et al. 2022 ^51^ | AE + RT | 6 | 2 | 90 | Yes | Yes |
| Onerup et al. 2022 ^52^ | IMT + AE | 3 | 7 | 30 | Yes | Yes |
| Woodfield et al. 2022 ^53^ | AE | 4 | 3.5 | 30 | Yes | Yes |

**Legend:** AE= aerobic exercise; RT=Resistance training; IMT=Inspiratory muscle training

| Comparison | Studies | Within-Study | Reporting | Indirectness | Imprecision | Heterogeneity | Incoherence | Rating | Reason(S) For Downgrading |
| --- | --- | --- | --- | --- | --- | --- | --- | --- | --- |
| AE vs NST | 5 | No concerns | Low risk | Some concerns | Major concerns | No concerns | Major concerns | Very low | ["Indirectness","Imprecision","Incoherence"] |
| AE + RT vs NST | 9 | No concerns | Low risk | No concerns | Major concerns | No concerns | Major concerns | Low | ["Imprecision","Incoherence"] |
| IMT vs NST | 2 | No concerns | Low risk | No concerns | Major concerns | No concerns | Major concerns | Low | ["Imprecision","Incoherence"] |
| IMT + AE vs NST | 3 | No concerns | Low risk | No concerns | Major concerns | No concerns | Major concerns | Low | ["Imprecision","Incoherence"] |
| IMT + AE + RT vs NST | 2 | Some concerns | Low risk | No concerns | Major concerns | No concerns | Major concerns | Very low | ["Within-study bias","Imprecision","Incoherence"] |
| RT vs NST | 1 | No concerns | Low risk | No concerns | Major concerns | No concerns | Major concerns | Low | ["Imprecision","Incoherence"] |

**Supplementary Table 2- Assessment of evidence certainty for overall morbidity**

**Legend:** AE= aerobic exercise; RT=Resistance training; IMT=Inspiratory muscle training; NST= no specific training

**Supplementary Table 3- NMA for major mobidity rate according to Clavien-Dindo classification (>II)**

| **Total studies: 10 RCT**  **Total Participants: 1312**  **Inconsistency (τ^2^): 0.001**  **Heterogeneity (I^2^): 0%**  **Test for I^2^ and τ^2^: p=0.423** | **OR**  **(95% CI)** | **Anticipated absolute effect^ (95% CrI)** | | | **Certainty of the evidence^$^** | **P-score** |
| --- | --- | --- | --- | --- | --- | --- |
|  |  | **with NST*** | **with intervention** | **Difference**  **(Minimal important difference = ±10)** |  |  |
| **NST** | Reference comparator | Reference comparator | Reference comparator | Reference comparator | Reference  Comparator | 0.69 |
| **AE + RT** | 0.99  (0.53 to 1.83) | 189 per 1000 | 187 per 1000 | 2 per 1000 fewer  (from 89 fewer to 157 more) | ⊕◯◯◯, Very Low  Whitin study-bias, Imprecision Incoherence | 0.67 |
| **RT** | 0.97  (0.40 to 2.35) | 189 per 1000 | 183 per 1000 | 6 per 1000 fewer  (from 113 fewer to 255 more) | ⊕⊕◯◯, Low  Imprecision, Incoherence | 0.67 |
| **IMT + AE** | 1.53  (1.08 to 2.18) | 189 per 1000 | 289 per 1000 | 100 per 1000 more  (from 15 more to 223 more) | ⊕⊕◯◯, Low  Incoherence, heterogeneity | 0.25 |
| **AE** | 2.81  (0.26 to 30.10) | 189 per 1000 | 531 per 1000 | 342 per 1000 more  (from 139 fewer to 5,292 more) | ⊕⊕◯◯, Low  Incoherence, heterogeneity | 0.23 |

**Legend**: OR=NMA estimates are reported as odds ratio; CI: confidence interval; ^= Anticipated absolute effect compares two risks by calculating the difference between the risk of the intervention group with the risk of the control group; the p-score represents the probability, without uncertainty, that the approach would be the best; *= the baseline morbidity rate was assumed to be those of control group; $= certainty in evidence according to GRADE working group: i)High quality - The true effect lies close to that of the estimate of the effect; ii) Moderate quality - The true effect is likely to be close to the estimate of the effect, but there is a possibility that it is substantially different; iii) Low quality -The true effect may be substantially different from the estimate of the effect; iv)Very low quality: The true effect is likely to be substantially different from the estimate of effect; NST= No Specific Training; AE= Aerobic exercise; IMT= Inspiratory muscle training; RT= Resistance Training.

**Supplementary Table 4- Assessment of evidence certainty for major morbidity**

| **Comparison** | **Studies** | **Within-study** | **Reporting** | **Indirectness** | **Imprecision** | **Heterogeneity** | **Incoherence** | **Rating** | **Reason(s) for downgrading** |
| --- | --- | --- | --- | --- | --- | --- | --- | --- | --- |
| **AE VS NST** | 1 | No concerns | Low risk | No concerns | Major concerns | No concerns | Major concerns | Low | ["Imprecision","Incoherence"] |
| **AE + RT VS NST** | 6 | Some concerns | Low risk | No concerns | Major concerns | No concerns | Major concerns | Very low | ["Within-study bias","Imprecision","Incoherence"] |
| **IMT + AE VS NST** | 2 | No concerns | Low risk | No concerns | No concerns | Major concerns | Major concerns | Low | ["Heterogeneity","Incoherence"] |
| **NST VS RT** | 1 | No concerns | Low risk | No concerns | Major concerns | No concerns | Major concerns | Low | ["Imprecision","Incoherence"] |

**Legend:** AE= aerobic exercise; RT=Resistance training; IMT=Inspiratory muscle training; NST= no specific training

| **Total studies: 25 RCT**  **Total Participants: 2674**  **Inconsistency (τ^2^): 0**  **Heterogeneity (I^2^): 0%**  **Test for I^2^ and τ^2^: p=0.986** | **OR**  **(95% CI)** | **Anticipated absolute effect^ (95% CrI)** | | | **Certainty of the evidence^$^** | **P-score** |
| --- | --- | --- | --- | --- | --- | --- |
|  |  | **with NST*** | **with intervention** | **Difference**  **(Minimal important difference = ±10)** |  |  |
| **NST** | Reference comparator | Reference comparator | Reference comparator | Reference comparator | Reference  Comparator | 0.61 |
| **RT** | 0.85  (0.18 to 3.98) | 14 per 1000 | 12 per 1000 | 2 per 1000 fewer  (from 11 fewer to 42 more) | ⊕⊕◯◯, Low  Imprecision, Incoherence | 0.61 |
| **AE** | 1.01  (0.10 to 10.67) | 14 per 1000 | 14 per 1000 | 0 per 1000  (from 13 fewer to 136 more) | ⊕◯◯◯, Very Low  Indirectness, Imprecision, Incoherence | 0.53 |
| **IMT + AE** | 1.05  (0.21 to 5.27) | 14 per 1000 | 15 per 1000 | 1 per 1000  (from 11 fewer to 60 more) | ⊕⊕◯◯, Low  Imprecision, Incoherence | 0.53 |
| **IMT + AE + RT** | 1.02  (0 to 514.84) | 14 per 1000 | 14 per 1000 | 0 per 1000  (from 14 fewer to 7,182 more) | ⊕◯◯◯, Very Low  within study bias, Imprecision, Incoherence | 0.51 |
| **IMT** | 1.38  (0.47 to 4.01) | 14 per 1000 | 19 per 1000 | 5 per 1000 more  (from 7 fewer to 42 more) | ⊕⊕◯◯, Low  Imprecision, Incoherence | 0.41 |
| **AE + RT** | 1.75  (0.29 to 10.65) | 14 per 1000 | 25 per 1000 | 11 per 1000 more  (from 10 fewer to 135 more) | ⊕⊕◯◯, Low  Imprecision, Incoherence | 0.35 |

**Supplementary Table 5- NMA for mortality rate**

**Legend**: OR=NMA estimates are reported as odds ratio; CI: confidence interval; ^= Anticipated absolute effect compares two risks by calculating the difference between the risk of the intervention group with the risk of the control group; the p-score represents the probability, without uncertainty, that the approach would be the best; *= the baseline morbidity rate was assumed to be those of control group; $= certainty in evidence according to GRADE working group: i)High quality - The true effect lies close to that of the estimate of the effect; ii) Moderate quality - The true effect is likely to be close to the estimate of the effect, but there is a possibility that it is substantially different; iii) Low quality -The true effect may be substantially different from the estimate of the effect; iv)Very low quality: The true effect is likely to be substantially different from the estimate of effect; NST= No Specific Training; AE= Aerobic exercise; IMT= Inspiratory muscle training; RT= Resistance Training.

**Supplementary Table 6- Assessment of evidence certainty for mortality**

| **Comparison** | **studies** | **Within-study** | **Reporting** | **Indirectness** | **Imprecision** | **Heterogeneity** | **Incoherence** | **Rating** | **Reason(s) for downgrading** |
| --- | --- | --- | --- | --- | --- | --- | --- | --- | --- |
| **AE vs NST** | 5 | No concerns | Low risk | Some concerns | Major concerns | No concerns | Major concerns | Very low | ["Indirectness","Imprecision","Incoherence"] |
| **AE + RT vs NST** | 9 | No concerns | Low risk | No concerns | Major concerns | No concerns | Major concerns | Low | ["Imprecision","Incoherence"] |
| **IMT vs NST** | 3 | No concerns | Low risk | No concerns | Major concerns | No concerns | Major concerns | Low | ["Imprecision","Incoherence"] |
| **IMT + AE vs NST** | 3 | No concerns | Low risk | No concerns | Major concerns | No concerns | Major concerns | Low | ["Imprecision","Incoherence"] |
| **IMT + AE + RT vs NST** | 2 | Some concerns | Low risk | No concerns | Major concerns | No concerns | Major concerns | Very low | ["Within-study bias","Imprecision","Incoherence"] |
| **RT vs NST** | 1 | No concerns | Low risk | No concerns | Major concerns | No concerns | Major concerns | Low | ["Imprecision","Incoherence"] |

**Legend:** AE= aerobic exercise; RT=Resistance training; IMT=Inspiratory muscle training; NST= no specific training

| **Comparison** | **Studies** | **Within-study** | **Reporting** | **Indirectness** | **Imprecision** | **Heterogeneity** | **Incoherence** | **Rating** | **Reason(s) for downgrading** |
| --- | --- | --- | --- | --- | --- | --- | --- | --- | --- |
| **AE vs NST** | 5 | No concerns | Low risk | No concerns | Some concerns | Some concerns | Major concerns | Very low | ["Imprecision","Heterogeneity","Incoherence"] |
| **AE + RT vs NST** | 7 | Some concerns | Low risk | Some concerns | Major concerns | No concerns | Major concerns | Very low | ["Within-study bias","Indirectness","Imprecision","Incoherence"] |
| **IMT vs NST** | 2 | No concerns | Low risk | No concerns | Major concerns | No concerns | Major concerns | Low | ["Imprecision","Incoherence"] |
| **IMT + AE vs NST** | 3 | No concerns | Low risk | No concerns | Major concerns | No concerns | Major concerns | Low | ["Imprecision","Incoherence"] |
| **IMT + AE + RT vs NST** | 2 | Some concerns | Low risk | Some concerns | Major concerns | No concerns | Major concerns | Very low | ["Within-study bias","Indirectness","Imprecision","Incoherence"] |
| **RT vs NST** | 1 | No concerns | Low risk | No concerns | Major concerns | No concerns | Major concerns | Low | ["Imprecision","Incoherence"] |

**Supplementary Table 7- Assessment of evidence certainty for the length of stay**

**Legend:** AE= aerobic exercise; RT=Resistance training; IMT=Inspiratory muscle training; NST= no specific training

**Supplementary Table 8- Assessment of evidence certainty for the pneumonia rate**

| **Comparison** | **Studies** | **Within-study** | **Reporting** | **Indirectness** | **Imprecision** | **Heterogeneity** | **Incoherence** | **Rating** | **Reason(s) for downgrading** |
| --- | --- | --- | --- | --- | --- | --- | --- | --- | --- |
| **AE vs NST** | 3 | No concerns | Low risk | Some concerns | Major concerns | No concerns | Major concerns | Very low | ["Indirectness","Imprecision","Incoherence"] |
| **AE + RT vs NST** | 5 | No concerns | Low risk | Some concerns | Major concerns | No concerns | Major concerns | Very low | ["Indirectness","Imprecision","Incoherence"] |
| **IMT vs NST** | 3 | No concerns | Low risk | No concerns | Major concerns | No concerns | Major concerns | Low | ["Imprecision","Incoherence"] |
| **IMT + AE vs NST** | 1 | No concerns | Low risk | No concerns | Major concerns | No concerns | Major concerns | Low | ["Imprecision","Incoherence"] |
| **IMT + AE + RT vs NST** | 2 | Some concerns | Low risk | No concerns | Major concerns | No concerns | Major concerns | Very low | ["Within-study bias","Imprecision","Incoherence"] |

**Legend:** AE= aerobic exercise; RT=Resistance training; IMT=Inspiratory muscle training; NST= no specific training

**Supplementary Figures**

**Supplementary Figure-1 PRISMA Flow-chart**

**Previous studies**

**Identification of new studies via databases and registers**

Records removed *before screening*:

Duplicate records removed (n = 404)

Studies included from previous version of review (n = 7)

Records identified from*:

- PubMed / MEDLINE (n =477)
- Embase (n=357)
- Cochrane (n=47)

Total n = 881

**Identification**

Records excluded**

(n = 373)

Total studies included in review

(n = 25)

Reports assessed for eligibility

(n = 104)

Reports sought for retrieval

(n = 104)

Records screened

(n = 477)

Reports not retrieved

(n = 0)

**Screening**

Reports excluded:

- Meta-analyses (n = 2)
- Not relevant to research question (n = 81)
- Unextractable data (n=3)

New studies included in review

(n = 18)

**Included**

**Supplementary Figure-2, panel A – Network geometry for major morbidity**

**
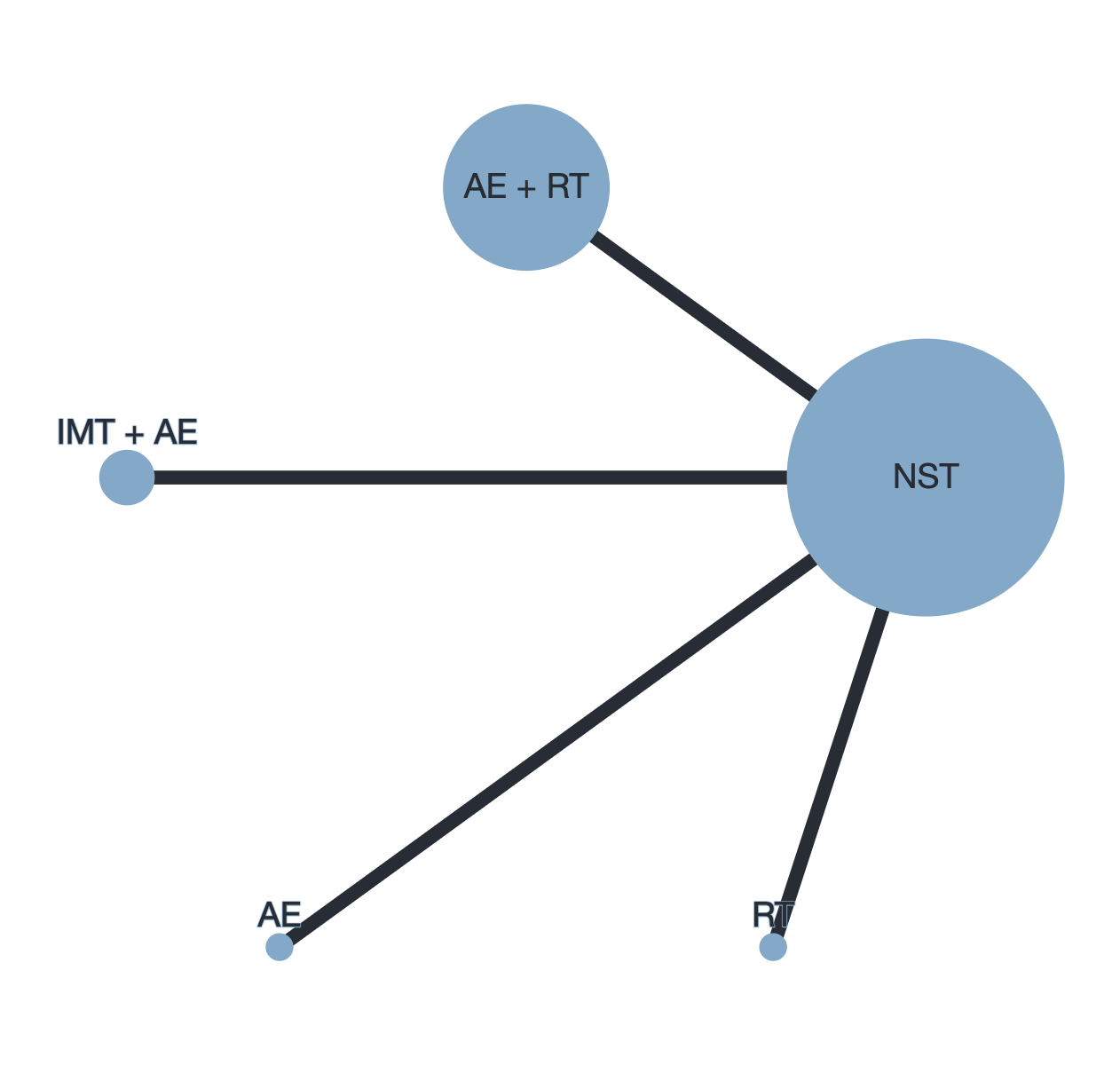
**

**Legend:** AE= aerobic exercise; RT=Resistance training; IMT=Inspiratory muscle training; NST= no specific training

**Supplementary Figure-2, panel B – Heat plot for major morbidity**

**Legend:** AE= aerobic exercise; RT=Resistance training; IMT=Inspiratory muscle training; NST= no specific training

**Supplementary Figure-2, panel C- Forest plot for major morbidity**

**Legend:** AE= aerobic exercise; RT=Resistance training; IMT=Inspiratory muscle training; NST= no specific training; OR= Odds ratio; P-score = the intervention is considered among the best if P-score was >= 0.66; when P-score was between 0.65 to 0.33, the combination was judged inferior to the best/better than the worst; when P-score was low than 0.33, the combination was considered among the worst.

**Supplementary Figure-2, panel D- Funnel plot for major morbidity**

**Legend:** AE= aerobic exercise; RT=Resistance training; IMT=Inspiratory muscle training; NST= no specific training

**Supplementary Figure-3, panel A- Network geometry for mortality**

**
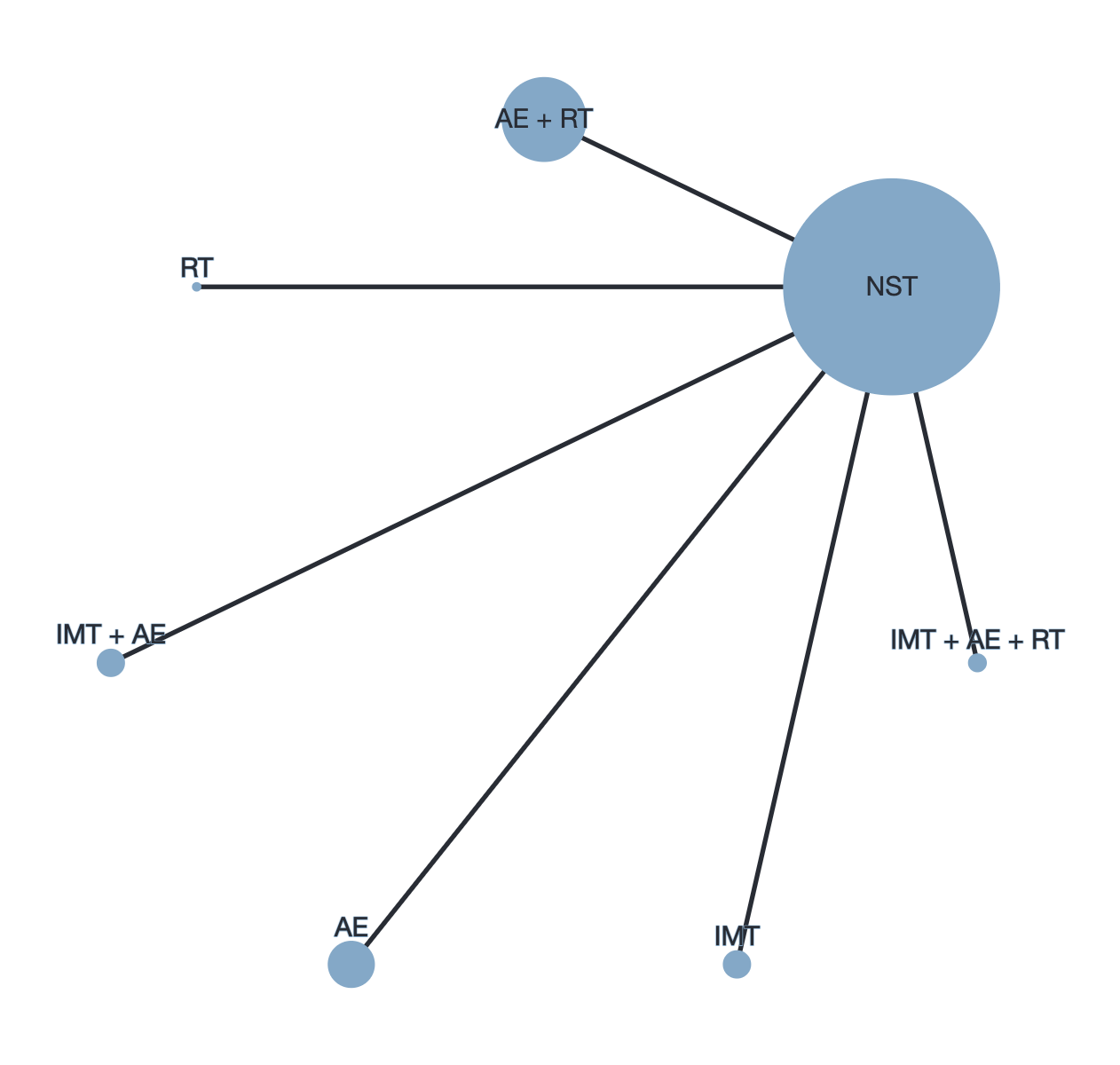
**

**Legend:** AE= aerobic exercise; RT=Resistance training; IMT=Inspiratory muscle training; NST= no specific training

**Supplementary Figure-3, panel B- Heat plot for mortality**

**Legend:** AE= aerobic exercise; RT=Resistance training; IMT=Inspiratory muscle training; NST= no specific training

**Supplementary Figure-3, panel C- Forest plot for mortality**

**Legend:** AE= aerobic exercise; RT=Resistance training; IMT=Inspiratory muscle training; NST= no specific training; OR= Odds ratio; P-score = the intervention is considered among the best if P-score was >= 0.66; when P-score was between 0.65 to 0.33, the combination was judged inferior to the best/better than the worst; when P-score was low than 0.33, the combination was considered among the worst.

**Supplementary Figure-3, panel D- Funnel plot for mortality**

**Legend:** AE= aerobic exercise; RT=Resistance training; IMT=Inspiratory muscle training; NST= no specific training

**Supplementary Figure-3, panel E- Density plot of component analysis**

**Legend:** AE= aerobic exercise; RT=Resistance training; IMT=Inspiratory muscle training; NST= no specific training; the component analysis showed the following incremental odds ratios: AE 1.09 (0.31; 3.87); IMT 1.22 (0.46; 3.22); RT 1.09 (0.31; 3.93); AE+IMT 1.34 (0.38; 4.62); AE+RT 1.19 (0.31; 4.66); IMT+RT 1.39 (0.24; 7.44); AE+IMT+RT 1.46 (0.34; 6.38)
